# Downregulation of NOX4 Expression by Roflumilast N-Oxide Reduces Markers of Fibrosis in Lung Fibroblasts

**DOI:** 10.1155/2013/745984

**Published:** 2013-08-21

**Authors:** Daniela Vecchio, Alessandra Acquaviva, Beatrice Arezzini, Hermann Tenor, Piero A. Martorana, Concetta Gardi

**Affiliations:** ^1^Department of Dermatology, Harvard Medical School, Wellman Center for Photomedicine, Massachusetts General Hospital, 40 Blossom Street, Boston, MA 02114, USA; ^2^Department of Physiopathology and Experimental Medicine, University of Siena, Via A. Moro 6, 53100 Siena, Italy; ^3^Takeda Pharmaceuticals International, 8152 Zurich, Switzerland

## Abstract

The phosphodiesterase 4 inhibitor roflumilast prevents bleomycin- (BLM-) induced lung fibrosis in animal models. However, its mechanism of action remains unknown. We investigated whether roflumilast N-oxide (RNO), the active metabolite of roflumilast, can modulate *in vitro* the oxidative effects of BLM on human lung fibroblasts (HLF). In addition, since BLM increases the production of F_2_-isoprostanes that have *per se* fibrogenic activity, the effect of RNO on oxidative stress and fibrogenesis induced by the F_2_-isoprostane 8-epi-PGF_2*α*_ was investigated. HLF were preincubated either with the vehicle or with RNO and exposed to either BLM or 8-epi-PGF_2*α*_. Proliferation and collagen synthesis were assessed as [^3^H]-thymidine and [^3^H]-proline incorporation. Reactive oxygen species (ROS) and F_2_-isoprostanes were measured. NADPH oxidase 4 (NOX4) protein and mRNA were also evaluated. BLM increased both cell proliferation and collagen synthesis and enhanced ROS and F_2_-isoprostane production. These effects were significantly prevented by RNO. Also, RNO significantly reduced the increase in both NOX4 mRNA and protein, induced by BLM. Finally, 8-epi-PGF_2*α*_ *per se* stimulated HLF proliferation, collagen synthesis, and NOX4 expression and ROS generation, and RNO prevented these effects. Thus, the antifibrotic effect of RNO observed *in vivo* may be related to its ability to mitigate ROS generation via downregulation of NOX4.

## 1. Introduction

Roflumilast is a selective phosphodiesterase 4 (PDE4) inhibitor approved for the treatment of severe chronic obstructive pulmonary disease (COPD) [1]. In experimental animals, roflumilast has been found to prevent cigarette smoke-induced emphysema [2] which is consistent with its clinical indication. However, it is of interest that in mice and rats challenged with the fibrogenic agent bleomycin (BLM), roflumilast was also found to reduce parameters of inflammation as well as markers of fibrosis such as lung collagen content and histologically assessed lung fibrosis [3]. Additionally, in these animals, the accumulation of lipid hydroperoxides in bronchoalveolar lavage fluid (BAL), a parameter of oxidative burden, was also attenuated by roflumilast. It was postulated that inhibition of either lung fibroblast function [4], the early inflammatory response [3], or the oxidative burden [5] induced by BLM may have contributed to the antifibrotic effect of roflumilast *in vivo.*

The present study was carried out *in vitro *on cultured human lung fibroblasts (HLF) to investigate a potential direct effect of roflumilast N-oxide (RNO), the active metabolite of roflumilast [6], on oxidative stress burden and fibrogenic effects induced by BLM.

Nicotinamide adenine dinucleotide phosphate oxidase 4 (NOX4) has been recognized as a major source of reactive oxygen species (ROS) and has recently been implicated in the fibrogenic response to lung injury [7, 8]. Lung fibroblasts from patients with idiopathic pulmonary fibrosis (IPF) express higher levels of NOX4 mRNA [9], and NOX4 siRNA has been found to mitigate the fibrotic response in BLM-treated mice [10]. Also, small molecule inhibitors of NOX4 have recently been reported to be effective in the BLM model [11]. RNO and other PDE4 inhibitors have previously been reported to significantly inhibit ROS production under experimental conditions [6], but no information is available on the effect of RNO on NOX4.

In addition, BLM was found to increase *in vivo *the production of F_2_-isoprostanes [12], the most proximal products of lipid peroxidation, which have been found to have fibrogenic activity *per se* [13, 14]. This suggests that F_2_-isoprostanes are not merely markers of oxidative stress but may also contribute to the pathogenesis of this condition. Thus, the effect of RNO on oxidative stress and markers of a fibrogenic process induced by the F_2_-isoprostane 8-epi-PGF_2*α*_ was also investigated.

## 2. Materials and Methods

### 2.1. HLF Treatment with BLM, 8-epi-PGF_2*α*_, and Roflumilast N-Oxide

Three different normal HLF (C-12360) were purchased from PromoCell GmbH (Heidelberg, Germany) and were originally obtained by never smokers donors. Selected experiments compared all three different HLF isolates. Cells were used between passages four and seven, seeded at a density of 6 × 10^4^ cells/mL in Dulbecco's modified Eagle's medium (DMEM) supplemented with 10% fetal bovine serum (FBS), and allowed to grow to confluence. Twenty-four hours before the treatment, the medium was changed to serum-free DMEM. Cells were preincubated for 30 min with vehicle (0.1% dimethyl sulfoxide) or RNO (Nycomed GmbH, Konstanz, Germany). RNO was used at 2 nM, corresponding to therapeutic plasma levels [15]. Cells were then treated with either BLM (Bleomycin Sulphate Nippon Kayaku, Sanofi Aventis) at 0.1–100 mU/mL or 8-epi-PGF_2*α*_ (BIOMOL Research Laboratories Inc., USA) in the range of concentrations 10^−10^–10^−7^ M previously detected in *in vivo* experiments [16]. A stock solution of 8-epi-PGF_2*α*_ (1 mg/mL in ethanol) was diluted to a concentration of 10^−5^ M and then further diluted to final concentrations with DMEM. Cytotoxicity was assessed by lactate dehydrogenase (LDH) Kit (DASIT S.p.A., Cornaredo, MI, Italy).

### 2.2. DNA Synthesis

Cell proliferation was evaluated in 24-well plates by measuring [^3^H]-thymidine incorporation, according to Boscoboinik et al. [17]. Subconfluent cells were incubated with either BLM or 8-epi-PGF_2*α*_ for 24 h in serum-free DMEM, in presence or absence of RNO. Six hours before the measurements, 10 *μ*Ci/mL of [^3^H]-thymidine was added. Cell layers were then washed, fixed for 20 min with ice-cold 5% trichloroacetic acid (TCA), and solubilized in 0.4 mL of 0.1 M NaOH/2% Na_2_CO_3_. Samples were mixed with 8 mL of Ultima Gold (Packard) and counted for radioactivity in a Packard 2100 TriCarb liquid scintillation analyser. Results are expressed as [^3^H]-thymidine incorporation (dpm) per well.

### 2.3. Collagen Synthesis Assay

To evaluate the effect of BLM or 8-epi-PGF_2*α*_ on collagen metabolism, HLF were seeded on 12-well plates and grown to visual confluence. Medium was changed to serum-free DMEM for 24 h to allow the cells to become relatively quiescent. In addition to DMEM, 11.5 *μ*g/mL L-proline, and 50 *μ*g/mL ascorbic acid, 50 *μ*g/mL *β*-aminopropionitrile were added. After incubation, cells were treated either with BLM or 8-epi-PGF_2*α*_ with or without RNO for 48 h. Collagen synthesis was assessed as previously reported [16]. Briefly, 16 h before the end of the 48 h treatment, 10 *μ*Ci/mL of [^3^H]-proline (Amersham International; specific activity 23 Ci/mmol) was added to each well. Media were harvested for determination of [^3^H]-proline incorporation into collagen and noncollagen proteins following the collagenase digestion method, by using highly purified bacterial collagenase (Calbiochem Cat. no. 234134, 250 IU). Incorporation of radioactivity into collagen and noncollagen proteins was determined following precipitation with TCA. Collagen-incorporated radioactivity was recovered in the TCA-soluble fraction, while noncollagen radioactivity was recovered from TCA precipitate. Percentage collagen synthesis was estimated as previously reported [18].

### 2.4. ROS Production

ROS levels were measured using the 2′,7′-dichlorodihydrofluorescein diacetate (H_2_DCFDA) probe as previously reported [19]. Prior to the experiments, cells were incubated in media containing 1% FBS, preincubated 30 min in presence or absence of RNO, and then treated with either BLM or 8-epi-PGF_2*α*_ for the indicated times. After washing, HLF were returned to Dulbecco's phosphate buffered saline (D-PBS) and loaded with 10 *μ*M H_2_DCFDA in D-PBS for 15 min at 37°C. Changes in fluorescence were measured by a Perkin-Elmer fluorescence plate reader.

### 2.5. Determination of 8-epi-PGF_2*α*_ Levels

HLF were seeded in T75 flasks (6 × 10^6^ cells/flask) and 8-epi-PGF_2*α*_ levels determined by enzyme immunoassay (Cayman Chemical, Ann Arbor, MI). F_2_-isoprostane levels are expressed as pg/10^6^ cells.

In order to investigate if NOX activity is involved in 8-epi-PGF_2*α*_ production, HLF were treated for 1 h with 1 *μ*M diphenyleneiodonium chloride (DPI) (Sigma-Aldrich), an NADPH oxidase inhibitor, before stimulation with 50 mU/mL BLM for 24 h, and then isoprostane levels were determined as described above.

### 2.6. NOX4 Expression in HLF

Protein lysates were collected, and Western blot analysis for NOX4 was performed using rabbit anti-human primary antibodies for NOX4 (1 : 1000, Novus Biologicals, Littleton, CO, USA) as previously described [20]. NOX4 mRNA expression was quantified by quantitative real-time RT-PCR by use of the PCR ABI Prism 7700 Sequence Detector (Perkin Elmer Applied Biosystems). Primers were synthesized using Primer Express version 1.0 software (PerkinElmer Applied Biosystems) according to the published cDNA sequences for NOX4 and *β*-actin as internal control.

### 2.7. Statistical Analyses

Each experiment was carried out a minimum of three times, and statistical analyses were performed. Bartlett's test was used to verify the equality of variance. Nonparametric Kruskal-Wallis test followed by Dunn's post-test was performed on data with unequal variances. When appropriate, one-way ANOVA and Tukey's post-test were used. A significant effect was indicated by a *P* value < 0.05. All data are presented as the mean ± SD for 3 independent experiments, each performed at least in triplicate.

## 3. Results

### 3.1. Effect of BLM on LDH Release from HLF

Bleomycin may trigger cytotoxicity and therefore LDH release from HLF was measured. Cells were treated for 24 h with BLM in the range of concentrations 0.1–100 mU/mL, as reported in Section 2. A significant increase in LDH release was observed only at the concentration of 100 mU/mL (data not shown). Thus, in the following studies, concentrations of BLM ≤50 mU/mL were used.

### 3.2. RNO Curbs BLM-Induced HLF Proliferation and Collagen Synthesis

**Figure 1:**
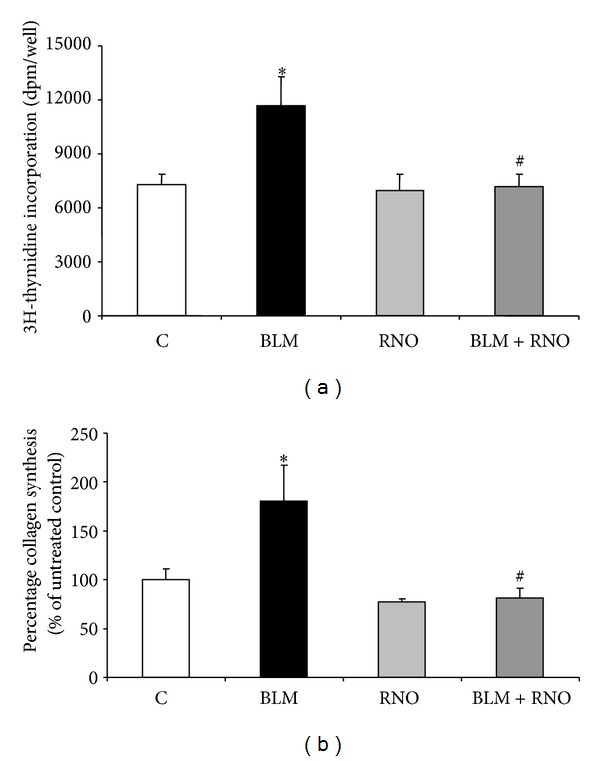
Effect of BLM and RNO on HLF proliferation and collagen synthesis. HLF were preincubated or not with RNO (2 nM) for 30 min and then treated with BLM (50 mU/mL). (a) Cell proliferation was evaluated on HLF from three different donors after 24 h by measuring [^3^H]-thymidine incorporation. Results are expressed as [^3^H]-thymidine incorporation (dpm) per well. Each sample was run in quadruplicate. Data are mean ± SD of 3 experiments. *P < 0.05 versus control (C); ^#^ *P* < 0.05 versus BLM. (b) For determination of [^3^H]-proline incorporation into collagen and noncollagen proteins, media were harvested after 48 h of treatment. Results are presented as percentage of untreated control. Data are mean ± SD of 3 experiments each in quadruplicate. *P < 0.05 versus control (C); ^#^ *P* < 0.05 versus BLM.

To evaluate the effect of RNO on the fibrogenic effects of BLM, HLF were preincubated with the PDE4 inhibitor and stimulated with 50 mU/mL of BLM. The addition of BLM-induced a significant increase in cell proliferation and collagen synthesis. [^3^H]-thymidine incorporation was increased by 1.6-fold (Figure 1(a)), while the relative collagen production, measured as percentage collagen synthesis over total protein synthesis, was increased by about 1.8-fold (Figure 1(b)). In unstimulated cells, RNO did not influence both parameters. However, when stimulated with BLM, RNO abolished the BLM-induced DNA and collagen synthesis in HLF (Figures 1(a) and 1(b)).

### 3.3. Effect RNO on Oxidative Stress Levels in HLF Treated with BLM

**Figure 2:**
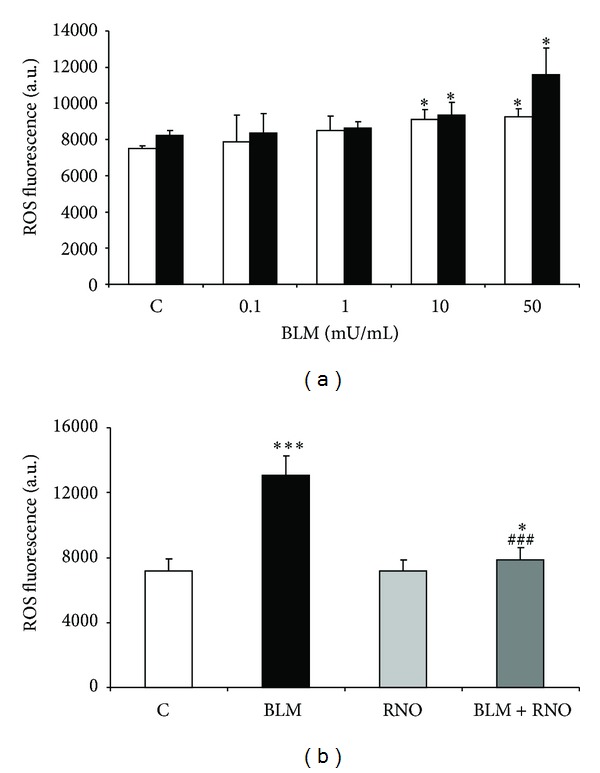
BLM-induced oxidative stress in HLF. (a) HLF were treated with various concentrations of BLM, and the generation of ROS was evaluated at 4 (white bar) and 24 (black bar) h. ROS levels were measured using the probe 2′,7′-dichlorodihydrofluorescein diacetate (H_2_DCFDA). ROS production was determined over a 60 min period. (b) The effect of RNO on the formation of ROS was evaluated in HLF from 3 different cell lines preincubated for 30 min in presence or absence of RNO (2 nM) and then treated with BLM (50 mU/mL) for 24 h. The results are expressed as arbitrary units of fluorescence measured on a PerkinElmer Applied Biosystems fluorescence plate reader (excitation 490 nm; emission 535 nm). Data are mean ± SD of 3 experiments. *P < 0.05 versus control (C); ***P < 0.001 versus control (C); ^###^ *P* < 0.001 versus BLM.

**Figure 3:**
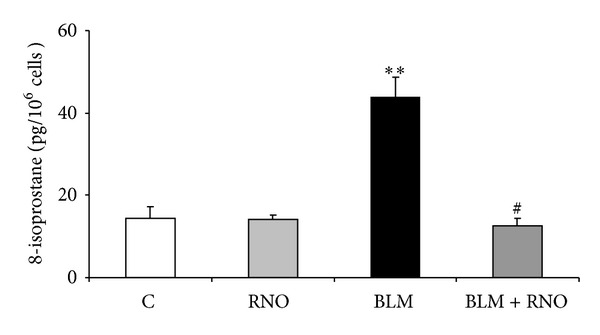
Effect of BLM and RNO on 8-epi-PGF_2*α*_ release in HLF. Levels of F_2_-isoprostanes (8-epi-PGF_2*α*_) were determined by an enzyme immunoassay kit in the media of HLF from 3 donors treated with BLM (50 mU/mL) for 24 h, in presence or absence of RNO (2 nM). Data are mean ± SD of 3 experiments. **P < 0.001 versus control (C); ^#^ *P* < 0.05 versus BLM.

**Figure 4:**
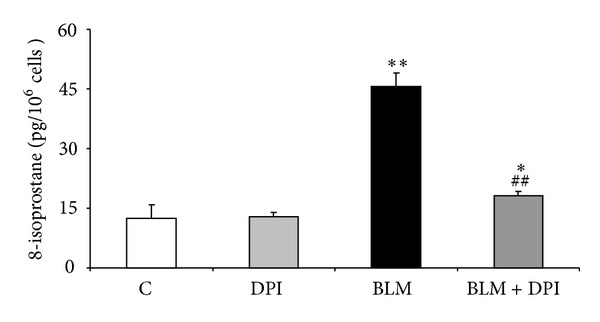
Involvement of NOX activity in BLM-induced 8-epi-PGF_2*α*_ release. Levels of F_2_-isoprostanes (8-epi-PGF_2*α*_) were determined in the media of HLF from 3 donors treated with BLM (50 mU/mL) for 24 h, in presence or absence of DPI (1 *μ*M), a broad NOX activity inhibitor. Data are mean ± SD of 3 separate experiments, each performed in triplicate. *P < 0.05 versus control (C); **P < 0.01 versus control (C); ^##^ *P* < 0.01 versus BLM.

To assess whether the reduced fibrogenic response (DNA and collagen synthesis) to BLM by RNO may be related to a decrease in oxidative burden, ROS release and generation of F_2_-isoprostanes were evaluated. For ROS determination, HLF were incubated for 4 and 24 h with BLM (0.1–50 mU/mL). An increased response was observed at both time intervals (Figure 2(a)). The effect of RNO on BLM-induced ROS release was evaluated after 24 h in HLF treated with or without 50 mU/mL BLM. In the absence of BLM, RNO did not affect ROS production. On the other hand, when stimulated with BLM, RNO reduced this increment by 87% (Figure 2(b)). The burden of oxidative stress was also evaluated as release of F_2_-isoprostanes. A 3-fold increase in 8-epi-PGF_2*α*_ production was observed in HLF treated with BLM (Figure 3). Pre-incubation with RNO abolished the BLM-induced F_2_-isoprostane increase. Furthermore, the presence of DPI (a broad spectrum NADPH oxidase inhibitor) strongly reduced the BLM-induced F_2_-isoprostane release in HLF (Figure 4).

### 3.4. Effect of BLM and RNO on NOX4 Expression in HLF

**Figure 5:**
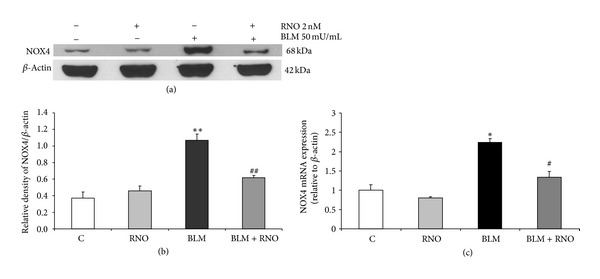
Effect of BLM and RNO on NOX4 expression in HLF. (a) Cells were preincubated or not with RNO (2 nM) and then treated for 24 h with BLM (50 mU/mL). Protein expression of NOX4 was analyzed by Western blotting. (b) Quantification of proteins was expressed as a ratio to *β*-actin. Data are mean ± SD of 3 experiments. **P < 0.01 versus control (C); ^##^ *P* < 0.01 versus BLM. (c) NOX4 gene expression in HLF was evaluated by real-time PCR. The isolated mRNA samples were analyzed using the specific primers and compared with *β*-actin (housekeeping gene) levels. Data are mean ± SD of 3 experiments. *P < 0.05 versus control (C); ^#^ *P* < 0.05 versus BLM.

A critical role of NOX4 in lung fibroblast ROS generation and fibrogenic signaling has been described, and NOX4 is increased in IPF lung fibroblasts [21]. Therefore, the effects of BLM and RNO on NOX4 expression were evaluated in HLF after an incubation period of 24 h. Immunoblot analysis revealed that BLM-induced about 3-fold increase in NOX4 protein (Figures 5(a) and 5(b)). No effect was seen with RNO in the absence of BLM. Pre-incubation with RNO prevented the NOX4 increase induced by BLM. A similar trend was observed for NOX4 mRNA expression (Figure 5(c)).

### 3.5. Effect of 8-epi-PGF_2*α*_ on LDH Release by HLF

The cytotoxicity of various 8-epi-PGF_2*α*_ concentrations was assessed by means of LDH release in HLF. After 24 h, a significant LDH release was observed only with the 10^−7^ M concentration (data not shown). 

### 3.6. Effect of 8-epi-PGF_2*α*_ and RNO on ROS Production and NOX4 Expression by HLF

**Figure 6:**
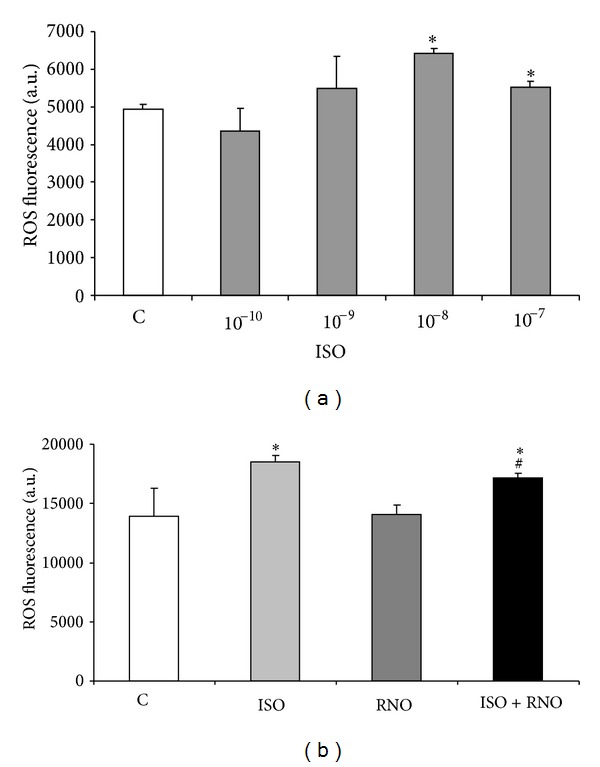
Effect of 8-epi-PGF_2*α*_ (ISO) and RNO on ROS production by HLF. (a) HLF were treated for 24 h with various concentrations of 8-epi-PGF_2*α*_. ROS levels were measured as described in Section 2.4. ROS production was determined over a 60 min period. (b) Cells were preincubated for 30 min in presence or absence of RNO (2 nM) and then treated with 8-epi-PGF_2*α*_ (10^−8 ^M) for 24 h. The results are expressed as arbitrary units of fluorescence. Data are mean ± SD of 3 experiments. *P < 0.05 versus control (C); ^#^ *P* < 0.05 versus 8-epi-PGF_2*α*_.

8-epi-PGF_2*α*_ induced an increase in ROS levels that peaked at 10^−8 ^M (1.3-fold, *P* < 0.05) following a 24 h incubation time. The increase in ROS observed with a concentration of 10^−7 ^M was lower than that observed with a concentration of 10^−8 ^M likely caused by cytotoxicity (Figure 6(a)). Thus, 8-epi-PGE_2*α*_ at 10^−8 ^M has been used in the following studies. RNO did not change ROS levels in resting HLF. On the other hand, pre-incubation with RNO significantly reduced the increase in ROS induced by 8-epi-PGF_2*α*_ (Figure 6(b)).

**Figure 7:**
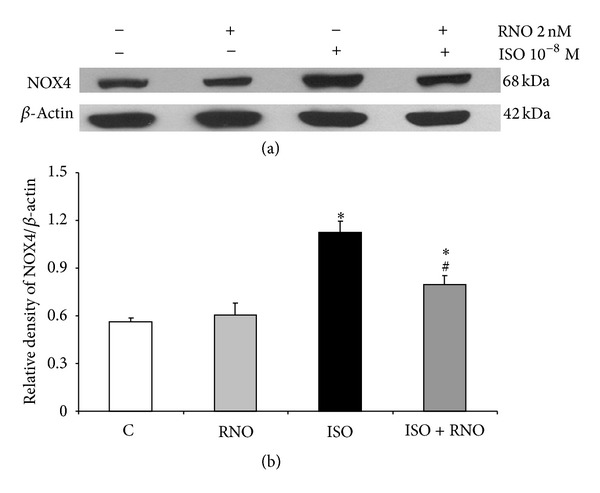
Effect of 8-epi-PGF_2*α*_ and RNO on NOX4 expression in HLF. Cells from 3 separate donors were preincubated or not with RNO (2 nM) and then treated for 24 h with 8-epi-PGF_2*α*_ (10^−8^ M). (a) Protein expression of NOX4 was analyzed by Western blotting. (b) Quantification of protein was expressed as a ratio to *β*-actin. Data are mean ± SD of 3 experiments. *P < 0.05 versus control (C); ^#^ *P* < 0.05 versus BLM.

Moreover, an increased expression of NOX4 was observed in HLF treated with 8-epi-PGE2_*α*_ for 24 h (Figures 7(a) and 7(b)), and this effect was prevented by the pre-incubation with RNO.

### 3.7. Effect of 8-epi-PGF_2*α*_ and RNO on HLF Proliferation and Collagen Synthesis

**Figure 8:**
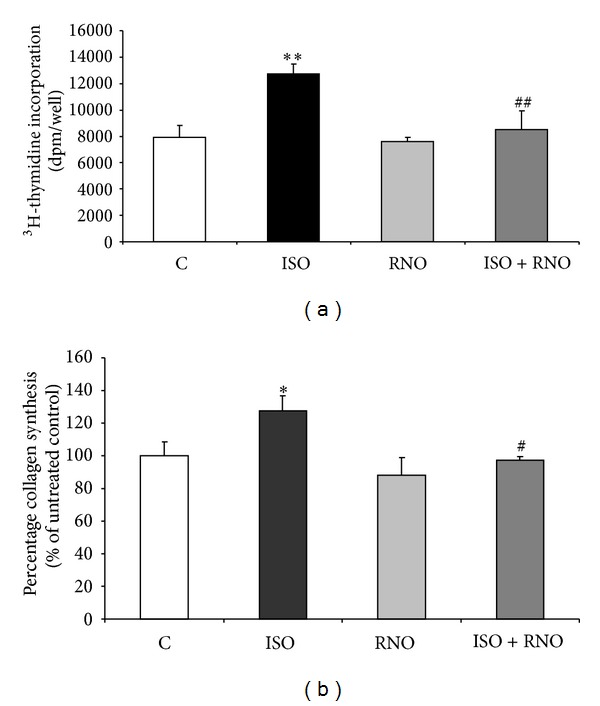
Effect of 8-epi-PGF_2*α*_ (ISO) and RNO on HLF proliferation and collagen synthesis. (a) Cells were preincubated for 30 min in presence or absence of RNO (2 nM) and then stimulated with 8-epi-PGF_2*α*_ (10^−8 ^M). Cell proliferation was evaluated after 24 h by measuring [^3^H]-thymidine incorporation in HLF. Results are expressed as [^3^H]-thymidine incorporation (dpm) per well. Each sample was run in quadruplicate. Data are mean ± SD of 3 experiments. **P < 0.01 versus control (C); ^##^ *P* < 0.01 versus 8-epi-PGF_2*α*_ (ISO). (b) Media were harvested after 48 h of treatment for determination of [^3^H]-proline incorporation into collagen and noncollagen proteins. Results (collagen/total protein) are presented as percentage of untreated control. Data are mean ± SD of 3 experiments. *P < 0.05 versus control (C); ^#^ *P* < 0.05 versus 8-epi-PGF_2*α*_ (ISO).

8-epi-PGF_2*α*_ (10^−8 ^M) significantly enhanced DNA synthesis. RNO abolished this 8-epi-PGF_2*α*_-induced increase in [^3^H]-thymidine incorporation in HLF. In the absence of 8-epi-PGF_2*α*_, RNO did not affect HLF proliferation (Figure 8(a)). 8-epi-PGF_2*α*_ also induced a moderate but significant increase in HLF collagen synthesis assessed as [^3^H]-proline incorporation after 48 h of incubation. In these experiments, the relative collagen production, measured as percentage collagen synthesis over total protein synthesis (collagenic plus noncollagenic proteins), was increased by about 1.3-fold. At baseline, RNO did not affect collagen production. However, when stimulated with 8-epi-PGF_2*α*_, pre-incubation with RNO prevented the 8-epi-PGF_2*α*_-induced collagen synthesis in HLF (Figure 8(b)).

## 4. Discussion

The main point of this study consists in the finding that the PDE4 inhibitor RNO prevented the oxidative stress induced by the fibrogenic agent BLM in HLF, and this was accompanied by a reduction of BLM-induced fibroblast proliferation and collagen release. In fact, RNO inhibited the BLM-induced ROS and F_2_-isoprostane production and abolished the increase in expression of NOX4 induced by BLM.

Recent studies indicate that NOX4 plays a role in the signaling pathways involved in pulmonary fibrosis pathophysiology [10, 21]. Stimulation of NOX4 expression was accompanied by an increase in ROS production [9]. Furthermore, NOX4-dependent production of H_2_O_2_ was required for myofibroblast differentiation and extracellular matrix production [10]. In an interesting* in vivo *study, BLM was administered to mice deficient in the p47^phox^ subunit of the NADPH oxidase complex. In this study, phorbol 12-myristate 13-acetate stimulation of BAL cells from KO mice produced no detectable ROS, while BAL cells from wild type mice did. In addition, hydroxyproline assays in the lung tissue at 14 days after BLM administration revealed the absence of collagen deposition in the lungs of the KO mice in contrast to wild type mice. The authors concluded that these findings provide strong evidence for cellular ROS production as an important component of the fibrogenic environment [22]. 

In the present study, BLM was found to increase NOX4 mRNA and protein expression and RNO was able to prevent this effect. To our knowledge, this is the first demonstration that RNO can modulate NOX4. Furthermore, we observed that BLM-induced a marked increase in 8-epi-PGF_2*α*_ production in HLF. It has been previously reported that 8-epi-PGF_2*α*_ induces NOX4 [23]. Accordingly, our data demonstrate that 8-epi-PGF_2*α*_ increased the formation of ROS and upregulated NOX4 expression in HLF. The presence of a broad inhibitor of NOX activity (DPI) decreased the BLM-induced isoprostane release in these cells.

Since in the present study RNO prevented both the BLM- and 8-epi-PGF_2*α*_-induced ROS production, it is suggested that this effect may be, at least in part, attributed to the inhibitory effect of RNO on NOX4.

In addition, in the current study, RNO inhibited BLM- and 8-epi-PGF_2*α*_-induced HLF proliferation and prevented the increase in HLF collagen synthesis induced by both BLM and 8-epi-PGF_2*α*_. Fibroblast proliferation and collagen synthesis are considered to play a pivotal role in the development of fibrosis. RNO has previously been reported to partially reduce [^3^H]-thymidine incorporation secondary to basic fibroblast growth factor in HLF [4]. It is conceivable that in our study the prevention by RNO of these BLM and 8-epi-PGF_2*α*_ effects may rest, at least in part, on its inhibition of ROS production by these two agents. It has been suggested that cAMP, or more specifically PDE4 inhibitors, can reduce ROS generation in human fibroblasts. Kokot et al. [24] demonstrated that the treatment with cAMP inducers suppressed BLM-induced expression of collagen I and III in human dermal fibroblasts.

F_2_-isoprostanes are considered as reliable markers of oxidative stress [25]. There is broad evidence that F_2_-isoprostanes are augmented in experimental animals after instillation of BLM [5, 12], indicating systemic oxidative stress. Recently, F_2_-isoprostanes have also been reported to trigger a fibrotic response. For example, in the liver, F_2_-isoprostanes were found to increase collagen deposition and cellular proliferation [13, 14]. These effects involve a modified form of isoprostane receptor, homologous to the classic thromboxane A2-binding receptor [14]. It was this fibrogenic activity of the F_2_-isoprostanes that prompted us to analyze the effect of RNO on 8-epi-PGF_2*α*_-stimulated HLF in the present study. While the fibrogenic response was confirmed in HLF, as an additional effect it was observed that 8-epi-PGF_2*α*_ increased both ROS production and NOX4 expression in these cells.

## 5. Conclusions

The results of the present study demonstrate that RNO had a powerful effect in preventing collagen deposition and fibroblast proliferation induced by both BLM and 8-epi-PGF_2*α*_. This was accompanied by an inhibitory effect on the expression of NOX4 and on the production of ROS and F_2_-isoprostanes.

In the context of previous reports, these findings may support the concept that the inhibition of the fibrogenic process may be related to the ability of the PDE4 inhibitor to mitigate cellular ROS generation. Further, these results may provide a rationale for the reduction of BLM-induced lung fibrosis by roflumilast *in vivo *in animal models.
